# Performance of established disease severity scores in predicting severe outcomes among adults hospitalized with influenza—FluSurv‐NET, 2017–2018

**DOI:** 10.1111/irv.13228

**Published:** 2023-12-17

**Authors:** Joshua D. Doyle, Shikha Garg, Alissa C. O'Halloran, Lauren Grant, Evan J. Anderson, Kyle P. Openo, Nisha B. Alden, Rachel Herlihy, James Meek, Kimberly Yousey‐Hindes, Maya L. Monroe, Sue Kim, Ruth Lynfield, Melissa McMahon, Alison Muse, Nancy Spina, Lourdes Irizarry, Salina Torres, Nancy M. Bennett, Maria A. Gaitan, Mary Hill, Charisse N. Cummings, Carrie Reed, William Schaffner, H. Keipp Talbot, Wesley H. Self, Derek Williams

**Affiliations:** ^1^ Influenza Division, National Center for Immunization and Respiratory Diseases, CDC Atlanta Georgia USA; ^2^ Epidemic Intelligence Service, CDC Atlanta Georgia USA; ^3^ Emory University School of Medicine Atlanta Georgia USA; ^4^ Atlanta Veterans Affairs Medical Center Atlanta Georgia USA; ^5^ Georgia Emerging Infections Program, Georgia Department of Health Atlanta Georgia USA; ^6^ Colorado Department of Public Health and Environment Denver Colorado USA; ^7^ Connecticut Emerging Infections Program Yale School of Public Health New Haven Connecticut USA; ^8^ Maryland Department of Health Baltimore Maryland USA; ^9^ Communicable Disease Division, Michigan Department of Health and Human Services Lansing Michigan USA; ^10^ Minnesota Department of Health Saint Paul Minnesota USA; ^11^ New York State Department of Health Albany New York USA; ^12^ New Mexico Department of Health Albuquerque New Mexico USA; ^13^ University of Rochester School of Medicine and Dentistry Rochester New York USA; ^14^ Salt Lake County Health Department Salt Lake City Utah USA; ^15^ Vanderbilt University School of Medicine Nashville Tennessee USA

**Keywords:** disease severity, hospitalization, influenza, mortality

## Abstract

**Background:**

Influenza is a substantial cause of annual morbidity and mortality; however, correctly identifying those patients at increased risk for severe disease is often challenging. Several severity indices have been developed; however, these scores have not been validated for use in patients with influenza. We evaluated the discrimination of three clinical disease severity scores in predicting severe influenza‐associated outcomes.

**Methods:**

We used data from the Influenza Hospitalization Surveillance Network to assess outcomes of patients hospitalized with influenza in the United States during the 2017–2018 influenza season. We computed patient scores at admission for three widely used disease severity scores: CURB‐65, Quick Sepsis‐Related Organ Failure Assessment (qSOFA), and the Pneumonia Severity Index (PSI). We then grouped patients with severe outcomes into four severity tiers, ranging from ICU admission to death, and calculated receiver operating characteristic (ROC) curves for each severity index in predicting these tiers of severe outcomes.

**Results:**

Among 8252 patients included in this study, we found that all tested severity scores had higher discrimination for more severe outcomes, including death, and poorer discrimination for less severe outcomes, such as ICU admission. We observed the highest discrimination for PSI against in‐hospital mortality, at 0.78.

**Conclusions:**

We observed low to moderate discrimination of all three scores in predicting severe outcomes among adults hospitalized with influenza. Given the substantial annual burden of influenza disease in the United States, identifying a prediction index for severe outcomes in adults requiring hospitalization with influenza would be beneficial for patient triage and clinical decision‐making.

## 1 INTRODUCTION

Seasonal influenza accounts for up to 140,000–810,000 hospitalizations per year in the United States [1]. Influenza can also result in more severe outcomes, including the need for intensive care unit (ICU) admission, advanced respiratory support, organ failure, and death [2]. The 2017–2018 influenza season was characterized as a high‐severity season [3], with the highest rates of influenza‐associated hospitalizations since the 2009 pandemic, and resulted in over 50,000 deaths in the United States [4]. Early identification of patients with influenza who are at increased risk of severe outcomes would help guide patient triage, early interventions, and other aspects of clinical decision‐making; however, correctly identifying those patients at increased risk for severe outcomes is challenging.

A number of severity scoring systems have been devised to objectively quantify clinical severity, incorporating a variety of clinical and laboratory data. Some, such as the Quick Sepsis Related Organ Failure Assessment (qSOFA), were designed to evaluate the risk of in‐hospital mortality among all hospitalized adults [5]. Others, including the CURB‐65 index and the Pneumonia Severity Index (PSI), were designed to assess the risk of mortality attributable to community‐acquired pneumonia, but have been used in patients hospitalized with respiratory viruses [6] and for helping differentiate patients with pneumonia who should be considered for hospitalization from those who should be considered for treatment as outpatients [7]. Although prior studies have evaluated the performance of several of these scores in patients hospitalized with influenza, most of these have been small and limited to single institutions. Validation of the performance of these scores in predicting severe outcomes among a broad and geographically diverse population of patients hospitalized with influenza would provide a useful tool for healthcare providers to appropriately triage patients and implement early interventions to decrease the morbidity and mortality attributable to influenza.

We used data from the CDC's Influenza Hospitalization Surveillance Network (FluSurv‐NET), a large, multi‐site, population‐based surveillance system representing ~9% of the US population, to evaluate the performance of existing severity scores in predicting severe outcomes among over 8000 adults hospitalized with laboratory‐confirmed influenza during the high‐severity 2017–2018 influenza season.

## 2 METHODS

### 2.1 Study population

We analyzed FluSurv‐NET data from patients 18 years of age and older hospitalized with laboratory‐confirmed influenza during the 2017–2018 influenza season. FluSurv‐NET has been previously described in detail [8, 9]. FluSurv‐NET conducts active, population‐based surveillance for influenza‐associated hospitalizations among residents of defined catchment areas who are hospitalized with laboratory‐confirmed influenza from October 1 to April 30 of each influenza season. FluSurv‐NET sites include select counties in California, Colorado, Connecticut, Georgia, Maryland, Michigan, Minnesota, New Mexico, New York, Ohio, Oregon, Tennessee, and Utah. Patients are included in FluSurv‐NET surveillance if they have a positive influenza laboratory test within 14 days prior to or anytime during hospital admission. Laboratory testing is ordered at clinician's discretion or based on facility testing practices and includes rapid antigen detection tests, reverse‐transcription polymerase chain reaction (RT‐PCR), immunofluorescence antibody staining, and viral culture. For this analysis, we included 11 FluSurv‐NET surveillance sites that collected additional data elements to characterize disease severity at hospital admission during the 2017–2018 influenza season.

Using a standardized case report form, trained surveillance staff conducted medical chart abstractions on hospitalized patients identified through FluSurv‐NET to obtain clinical information on underlying conditions, hospital course, and outcomes, including ICU admission, invasive mechanical ventilation, extracorporeal membrane oxygenation (ECMO), and in‐hospital death. A supplemental disease severity case report form (Supplemental Figure 1) was created for this project to collect information on vital signs, clinical signs and symptoms, and selected laboratory values obtained within one calendar day of hospital admission. Additional outcome data collected as part of the disease severity project included the use of non‐invasive respiratory support (continuous positive airway pressure [CPAP], bi‐level positive airway pressure [BiPAP], and high‐flow nasal cannula [HFNC]), and administration of vasopressor medications (dobutamine, dopamine, ephedrine, epinephrine, levophed, midodrine, milrinone, norepinephrine, neosynephrine, phenylephrine, and vasopressin).

A minimum set of variables required to generate age‐stratified rates of laboratory‐confirmed influenza‐associated hospitalizations was collected for all FluSurv‐NET patients; however, due to the large number of influenza‐related hospitalizations in older adults during the 2017–2018 season, a sampling protocol was implemented for standard medical chart abstraction for patients aged 50 years and older. Standard medical chart abstractions were conducted for all patients aged 18–49 years. Surveillance sites had the option of selecting 100% or a random sample of 50% or 25% of patients aged greater than 65 years, and 100% or a 50% random sample of patients aged 50 to 64 years. Sites were also given the option to select cases for inclusion in this disease severity project: some sites collected disease severity data on all patients within the surveillance catchment area, whereas others employed different approaches such as collecting disease severity data on all patients admitted to one or more selected hospitals (Supplemental Table 1). For this analysis, we excluded patients with hospital‐acquired influenza (first positive influenza test ≥3 days after admission), as well as those with missing or implausible vital signs data and missing data on severe outcomes.

FluSurv‐NET sites obtained human subjects and ethics approvals from their respective state health department and academic partner Institutional Review Boards (IRBs) as needed. CDC determined this activity met the requirement for public health surveillance; therefore, the CDC's IRB approval was not required.

### 2.2 Data analysis

Data were weighted to reflect the probability of selection for medical chart abstraction as part of standard FluSurv‐NET surveillance. Sample sizes throughout are listed as unweighted values (*n*), while frequency percentages, sensitivities, and specificities are listed as weighted values. Data were not weighted to reflect the probability of inclusion of cases in the disease severity project (which required completion of the supplemental disease severity case report form) because inclusion criteria varied across sites. We described the characteristics of our study population as well as the distribution of individual outcomes. For each patient, we calculated values at the time of hospital admission for three disease severity classification scores: pneumonia severity index (PSI), quick Sepsis Related Organ Failure Assessment (qSOFA), and CURB‐65 (Supplemental Table 2). We used the first available vital signs, laboratory values, and symptom documentation at the time of presentation to the emergency department or hospital for the calculation of severity score values. Of note, information regarding the presence or absence of pleural effusion (a component of PSI) was not collected during the 2017–2018 season, so we adjusted the thresholds for each PSI level downward by 10 points. For patients with missing laboratory values, we assumed that clinical suspicion of laboratory derangements was not sufficiently high to obtain the test and thus set missing values to normal as previously described [10].

We calculated receiver operating characteristic (ROC) curves for each clinical score against defined outcome tiers using unadjusted logistic regression, starting with the most severe outcome (death), then sequentially adding additional outcome tiers in decreasing order of severity: Tier 1 − in‐hospital death, Tier 2 − Tier 1 + mechanical ventilation or ECMO or vasopressors, Tier 3 − Tier 2 + CPAP or BIPAP or HFNC, and Tier 4 − Tier 3 + ICU admission. We also calculated the area under the ROC curve (AUC) for each pair of clinical scores and outcome tiers using unadjusted logistic regression, as an overall measure of discrimination.

We assigned patients to dichotomized high‐ or low‐risk groups for each severity score based on their distribution in our data set as well as published literature [5, 6, 11, 12]. We then calculated sensitivity, specificity, and odds ratios, with 95% confidence intervals, for each dichotomized risk group against the four defined outcome tiers. The odds ratios reflected the odds of having a particular outcome tier for patients assigned to a high‐risk group, in comparison with a low‐risk group for each of the three severity scores. Confidence intervals were generated in SUDAAN using the default Taylor Series linearization method for variance estimation to account for the complex sample design.

Statistical analyses were performed in SAS® version 9.4 (SAS Institute, Cary, NC, USA) and SUDAAN® version 11.0.3 (RTI International, Research Triangle Park, NC, USA).

## 3 RESULTS

### 3.1 Study population

**FIGURE 1:**
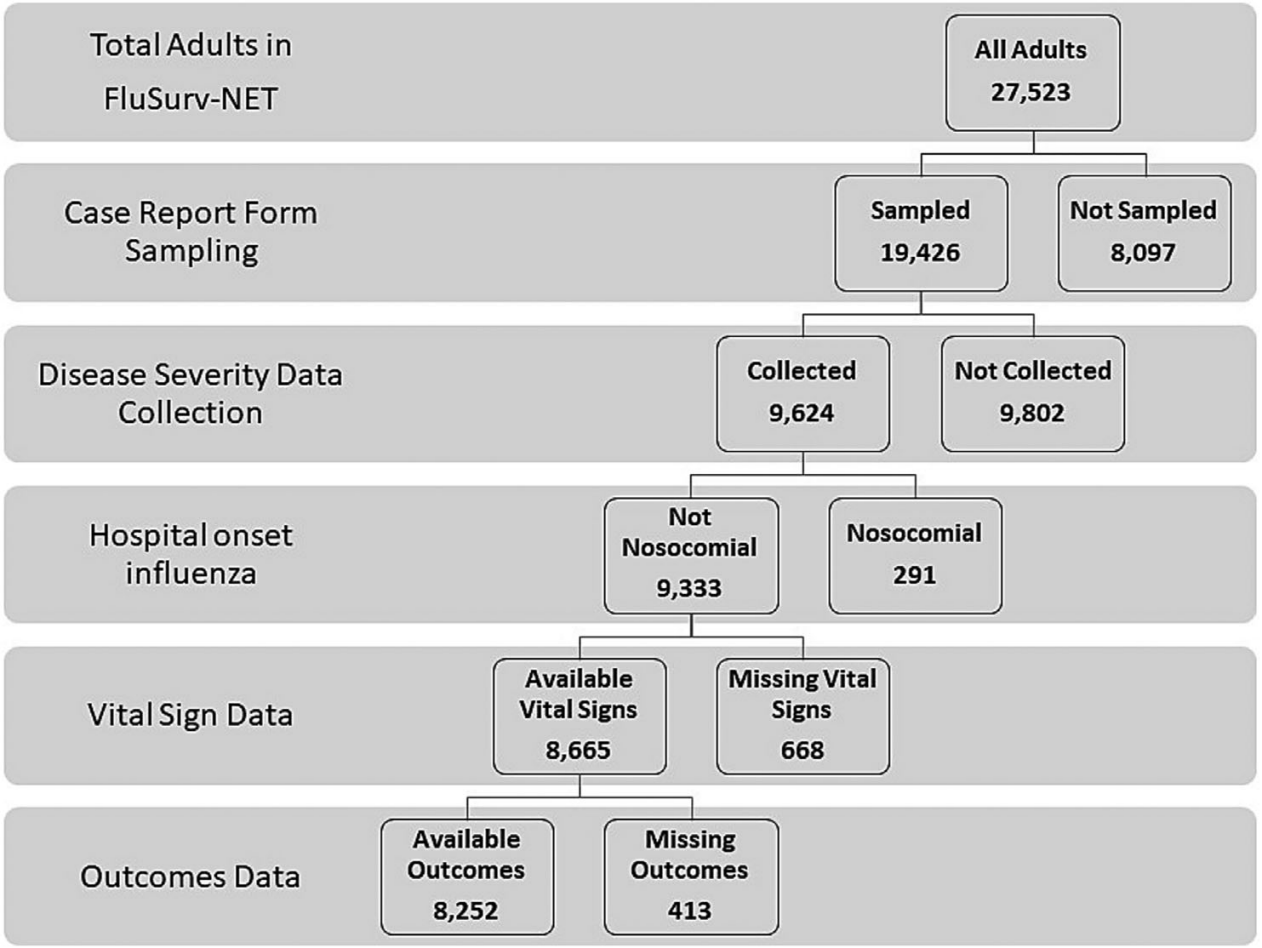
**Selection of cases for analysis of influenza‐associated hospitalizations, FluSurv‐NET, 2017–2018.** This schematic defines the strategy used for the inclusion of cases for analysis of influenza disease severity. There were 27,523 adults hospitalized with influenza in the FluSurv‐NET network during the 2017–2018 influenza season. A portion of patients were sampled for inclusion in this analysis, and case report forms were completed for a selection of these patients. Patients were subsequently excluded if they were determined to have developed a nosocomial influenza infection, if vital sign data were missing, or if outcome data was absent, yielding a final analytic dataset of 8252 individuals.

There were 27,523 cases of adult influenza hospitalizations identified through FluSurv‐NET during the 2017–2018 influenza season (Figure 1). Of these, 19,426 cases were sampled for complete medical record chart abstraction, and of those, 9624 cases were selected for collection of disease severity data (Supplemental Table 1). Of these individuals, 291 were determined to have hospital‐onset influenza infection, 668 had missing or implausible vital sign data, and 413 had missing outcome data; these individuals were excluded. After exclusions, 8252 individuals were included in this analysis. Characteristics of all FluSurv‐NET cases versus those included in this analysis are described in Supplemental Table 3.

### 3.2 Demographic and clinical characteristics

Among the analytic population, the median age was 71 years (IQR: 58–82) (Table 1). The majority of patients were male (57.0%); 64.9% were non‐Hispanic White, 21.6% non‐Hispanic Black, and 7.1% were Hispanic or Latino. A high proportion of patients (92.1%) had at least one underlying medical condition. The most common underlying medical conditions were cardiovascular disease (51.6%), chronic metabolic disease (45.4%), chronic lung disease excluding asthma (29.8%), and chronic renal disease (19.9%). A total of 36.9% of patients had obesity or extreme obesity, and 52.2% of patients had documented current or former tobacco use. Among patients, 49.6% had documented receipt of influenza vaccine (at least 14 days prior to hospital admission) during the current influenza season, and 92.8% of patients received antiviral treatment during hospitalization. The median length of hospital stay was 3 days (IQR 2 to 5).

**TABLE 1:** Demographic and clinical characteristics of adult patients hospitalized with influenza, FluSurv‐NET, 2017–2018.

	Unweighted n	Weighted %
**All, no. (%)**	8252	
**Age at enrollment, years**		
18–49	1301	13.8
50–64	1985	22.0
65+	4966	64.2
Median age, years (IQR)	8252	71 (58–82)
**Sex**		
Female	3554	43.0
Male	4698	57.0
**Race and ethnicity** ^a^		
Non‐Hispanic White	5354	64.9
Non‐Hispanic Black	1877	21.6
Non‐Hispanic American Indian or Alaskan Native	31	0.6
Non‐Hispanic Asian/Pacific Islander	161	2.0
Hispanic or Latino	490	7.1
**Network Site**		
Colorado	349	3.7
Connecticut	622	6.6
Georgia	108	2.3
Maryland	2254	23.9
Michigan	1130	12.0
Minnesota	740	13.1
New Mexico	199	6.8
New York—Albany	109	2.5
New York—Rochester	752	8.0
Tennessee	1211	12.9
Utah	778	8.3
**Residence at Hospital Admission** ^b^		
Private residence/assisted living/group home	7503	91.2
Nursing home/SNF/LTACH/rehab/hospice	513	6.2
Alcohol or drug abuse treatment/jail/mental hospital/homeless/other	226	2.6
**Presence of chronic medical conditions** ^c^		
0	640	7.6
1 or more	7576	92.1
**Chronic medical condition categories**		
Asthma	1658	19.8
Chronic lung disease (excluding asthma)	2391	29.8
Chronic metabolic disease	3633	45.4
Blood disorders/hemoglobinopathy	213	2.5
Cardiovascular disease	4163	51.6
Neuromuscular disorder	403	4.7
Neurologic disorder	1591	19.5
History of Guillain Barre syndrome	13	0.2
Immunosuppressive condition	1577	18.6
Renal disease	1567	19.9
Liver disease	429	5.1
**Weight categories** ^d^		
Not obese	5126	63.1
Obese	3122	36.9
Extreme obese	822	9.4
**Smoking status**		
Current	1730	19.6
Former	2599	32.6
No/unknown	3923	47.8
**Pregnant at admission** ^e^		
Yes	137	22.9
No/unknown	462	77.1
**Antiviral administration** ^f^		
Yes	7642	92.8
**Current season influenza vaccination** ^g^		
Yes	3914	49.6
No	2688	31.0
Unknown	1650	19.5
**Diagnosed with pneumonia** ^h^		
Yes	1697	20.5
**Days from symptom onset to admission** ^i^		
0–2 days	2687	33.4
3–4 days	2277	27.8
5–14 days	2028	23.9
**Median length of hospital stay in days (IQR)**	8252	3 (2–5)

^a^ Multiracial (unweighted *n* = 7; weighted percentage = 0.1%); Unknown race/ethnicity (unweighted *n* = 332; weighted percentage = 3.8%);

^b^ Unknown residence at hospital admission (unweighted *n* = 10; weighted percentage = 0.0%);

^c^ Underlying Conditions unknown (unweighted *n* = 36; weighted percentage = 0.4%);

^d^ Weight categories defined as follows: BMI < 30 = not obese, BMI ≥ 30 = obese, BMI ≥ 40 = Extreme obesity; Unknown obesity (unweighted *n* = 4; weighted percentage = 0.1%);

^e^ Pregnancy status ascertained among women aged 18–44 years;

^f^ Antivirals included oseltamivir, peramivir, and baloxavir; unknown antiviral prescription (unweighted *n* = 13, weighted percentage = 0.1%);

^g^ Vaccination status defined as receipt of current season vaccination at least 14 days prior to influenza‐positive laboratory test;

^h^ Pneumonia defined as a combination of radiographic findings of bronchopneumonia, air space opacity, consolidation, lobar or interstitial infiltrate within 3 days of hospital admission, and either an ICD‐coded discharge diagnosis of pneumonia or documentation of pneumonia on hospital discharge summary;

^i^ Unknown or >14 days from symptom onset to admission (unweighted *n* = 1260; weighted percentage = 14.9%).

### 3.3 Outcome frequencies

Among 8252 included patients, we characterized the type and frequency of outcomes (non‐mutually exclusive) experienced during the course of hospitalization (Table 2A). We grouped patients into four outcome tiers, in decreasing order of severity, with each tier becoming increasingly more inclusive of outcomes (Table 2B). The first outcome tier (individuals who died during hospitalization) included 1.9% of patients, the second tier (patients who died during hospitalization or required vasopressors or invasive mechanical ventilation or ECMO) included 5.8% of patients, the third tier (patients who died during hospitalization or required vasopressors or invasive mechanical ventilation or ECMO or CPAP or BiPAP or HFNC) included 19.8% of patients, and the fourth tier (patients who died during hospitalization or required vasopressors or invasive mechanical ventilation or ECMO or CPAP or BiPAP or HFNC or ICU admission) included 24.2% of patients.

**TABLE 2A:** Frequency of non‐mutually exclusive outcomes among adults hospitalized with influenza, FluSurv‐NET, 2017–2018.

Outcomes^a^	Total (*n*)	Weighted %
	8252	
Death during hospitalization	183	1.9%
Vasopressor administration	293	3.4%
Mechanical ventilation	336	3.8%
Extracorporeal membrane oxygenation	9	0.1%
Continuous positive airway pressure	490	6.2%
Bi‐level positive airway pressure	777	9.2%
High‐flow nasal cannula	603	8.2%
Intensive care unit admission	1127	13.3%

*Note*: Non‐mutually exclusive severe patient outcomes are presented as unweighted numbers (*n*) and as a percentage of total patients, weighted by probability of selection.

^a^ Number and weighted percentage of patients with no “severe” outcomes of interest: 6319/8252 (75.8%).

**TABLE 2B:** Distribution of severe outcome tiers among adults hospitalized with influenza, FluSurv‐NET, 2017–2018.

Outcome tiers	Total (*n*)	Weighted %
	8252	
Tier 1: Death	183	1.9
Tier 2: Tier 1 plus vasopressors or mechanical ventilation or extracorporeal membrane oxygenation	514	5.8
Tier 3: Tier 2 plus continuous positive airway pressure or bi‐level positive airway pressure or high‐flow nasal cannula	1571	19.8
Tier 4: Tier 3 plus intensive care unit admission	1933	24.2

*Note*: Patients with severe outcomes are grouped into four progressively more inclusive tiers. Patients who died during hospitalization were grouped into Tier 1; those who died during hospitalization or who required invasive respiratory support or vasopressors were grouped into Tier 2; those in Tier 1, Tier 2, or who required non‐invasive respiratory support were grouped into Tier 3; and those in Tier 1, Tier 2, Tier 3, or who required ICU admission were grouped into Tier 4. Data are presented as unweighted numbers (*n*) and as a percentage of total patients, weighted by probability of selection.

### 3.4 Disease severity scores

The distribution of the three disease severity scores, PSI, qSOFA, and CURB‐65, among the 8252 patients are shown in Table 3. We classified patients into dichotomized high‐ or low‐risk groups based on each score, taking into account the distribution of each score in our dataset as well as published literature using dichotomized severity scores as described in the Methods section. For PSI, patients with a score placing them in Class V were considered high‐risk; 15.3% fell into this group. For qSOFA, patients were classified as high‐risk if they had a score of 2 or 3; 7.4% of patients fell into this group. Finally, for CURB‐65, patients with scores of 3, 4, or 5 were considered high risk; 17% fell into this group.

**TABLE 3:** Distribution of clinical severity scores and binary risk thresholds among adults hospitalized with influenza, FluSurv‐NET, 2017–2018.

Severity scores	Total (*n*)	Weighted %	Risk group	Number in risk group	Weighted % in risk group
	8252				
**PSI**					
I	487	5.2%	Low	7049	84.7
II	1285	14.3%			
III	1828	21.8%			
IV	3449	43.4%			
V	1203	15.3%	High	1203	15.3
**QSOFA**					
0	4408	52.8%	Low	7659	92.6
1	3251	39.8%			
2	557	7.0%	High	593	7.4
3	36	0.4%			
**CURB‐65**					
0	1929	21.0%	Low	6932	83.0
1	2620	31.7%			
2	2383	30.3%			
3	1104	14.1%	High	1320	17.0
4	209	2.8%			
5	7	0.1%			

*Note*: Patients were evaluated based on PSI, QSOFA, and CURB‐65 criteria at the time of presentation and grouped by score. Each clinical score was also dichotomized into a “low”‐ and “high”‐risk group. Data are presented as unweighted numbers (*n*) and as a percentage of total patients, weighted by probability of selection.

### 3.5 ROC curves and sensitivity, specificity, and odds ratios

**FIGURE 2:**
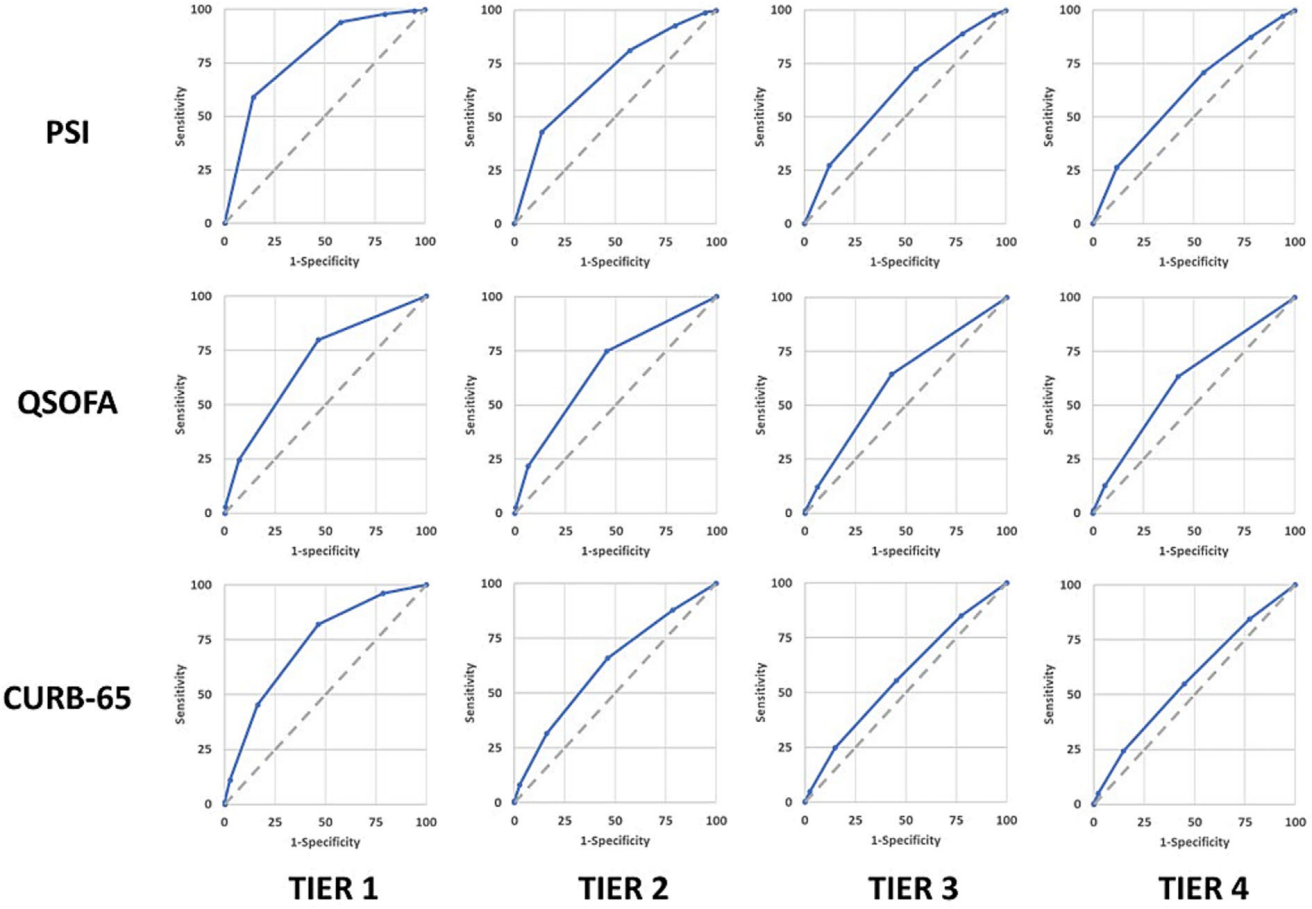
**Receiver‐operator characteristic curves for PSI, QSOFA, and CURB‐65 against selected outcome tiers, FluSurv‐NET, 2017–2018.** The receiver‐operator characteristic (ROC) curves for PSI, QSOFA, and CURB‐65 are plotted against patients who experienced four tiers of severe outcomes. Tier 1 includes patients whose disease resulted in in‐hospital mortality; Tier 2 includes those patients in Tier 1 as well as those patients who required vasopressors, invasive mechanical ventilation, or extracorporeal membrane oxygenation (ECMO); Tier 3 includes patients in Tier 2 as well as patients who required non‐invasive respiratory support (CPAP, BiPAP, or HFNC); Tier 4 includes patients in Tier 3 as well as patients who required admission to the intensive care unit (ICU).

ROC curves for PSI, qSOFA, and CURB‐65 against each of the four severity outcome tiers are depicted in Figure 2. The sensitivity, specificity, and odds ratios for each dichotomized (i.e., stratified into high and low risk) severity score against each outcome tier, along with 95% confidence intervals, are shown in Table 4. In general, all severity scores demonstrated lower sensitivity than specificity for the outcomes tested. Sensitivity estimates were highest for death. For instance, PSI had a sensitivity of 59% and specificity of 86% for death, qSOFA had a sensitivity of 25% and specificity of 93% for death, and CURB‐65 had a sensitivity of 45% and specificity of 84% for death. Overall, the discrimination (AUC) for each score was lower for less severe outcomes and higher for more severe outcomes. The best discrimination (0.78) was for PSI against death.

**TABLE 4:** Performance of disease severity scores in predicting severe outcome tiers among adults hospitalized with influenza, FluSurv‐NET, 2017–2018.

	Outcome tiers	Unweighted n	Sensitivity (%)	Sensitivity	Specificity (%)	Specificity	OR	OR 95% CI	AUC
				95% CI		95% CI			
**PSI** ^a^	Tier 1	183	59.0	52.0–66.0	85.6	84.8–86.4	8.6	6.4–11.5	0.78
	Tier 2	514	43.2	38.7–47.7	86.5	85.6–87.3	4.8	4.0–5.9	0.69
	Tier 3	1571	27.4	24.9–29.8	87.7	86.8–88.6	2.7	2.3–3.1	0.62
	Tier 4	1933	26.4	24.2–28.6	88.3	87.4–89.2	2.7	2.3–3.1	0.62
**QSOFA** ^a^	Tier 1	183	24.6	18.8–30.3	92.9	92.3–93.6	4.3	3.1–6.0	0.70
	Tier 2	514	21.7	18.2–25.3	93.5	92.9–94.1	4.0	3.2–5.0	0.67
	Tier 3	1571	12.1	10.4–13.8	93.8	93.1–94.4	2.1	1.7–2.5	0.61
	Tier 4	1933	12.7	11.1–14.3	94.3	93.6–95.0	2.4	2.0–2.9	0.61
**CURB‐65** ^a^	Tier 1	183	45.4	38.5–52.2	83.6	82.6–84.5	4.3	3.2–5.6	0.73
	Tier 2	514	31.7	27.6–35.8	83.9	83.0–84.9	2.4	2.0–3.0	0.63
	Tier 3	1571	24.9	22.5–27.3	85.0	84.0–86.0	1.9	1.6–2.2	0.58
	Tier 4	1933	24.2	22.0–26.4	85.3	84.3–86.3	1.9	1.6–2.1	0.57

*Note*: The performance of dichotomized PSI, QSOFA, and CURB‐65 scores was determined against patients in each tier of severe outcomes. The sensitivity, specificity, specificity, and OR of each score are shown, along with 95% confidence intervals. The area under the receiver‐operator curve (AUC) is also shown for the three clinical scores against the four defined tiers of severe outcomes.

^a^ Calculations based on dichotomized (high/low risk) severity scores (PSI: low risk = I‐IV, high risk = V; QSOFA: low risk = 0,1; high risk = 2,3; CURB‐65: low risk = 0–2, high risk = 3–5).

## 4 DISCUSSION

We evaluated the performance of three established disease severity scores in predicting severe influenza‐related outcomes among a population‐based surveillance network of adults hospitalized with laboratory‐confirmed influenza in the United States during the 2017–2018 influenza season, which was predominated by influenza A(H3N2) viruses in the United States. Among the 8252 adult patients included in our analysis, all three severity scores had better discrimination for more severe outcomes, such as death, and worse discrimination for less severe outcomes such as ICU admission alone. For instance, we observed the highest discrimination, at 0.78, for the PSI against in‐hospital mortality, perhaps expectedly as all three of these scores were initially developed to predict mortality. By comparison, PSI was reported to have a discrimination of 0.83 for death in the initial validation cohort of hospitalized patients with community‐acquired pneumonia [7], and the initial validation of the qSOFA score determined an AUROC of 0.66 for mortality in ICU patients and 0.81 in hospitalized patients [5]. Assigning participants to dichotomized high‐ and low‐risk groups further enriches the high‐risk groups in our study with individuals at greater risk of in‐hospital mortality.

Previous studies have assessed the performance of scores in predicting outcomes related to respiratory viruses, including influenza, reviewed by Adams et al. [13]. In general, indices validated against community‐acquired pneumonia have demonstrated poor discrimination (ROC area ≤ 0.7) for influenza‐related outcomes, including the need for hospital admission [14], need for a higher level of care [12], ICU admission [15], and mortality [16, 17, 18, 19, 20]. Interestingly, the PSI has been previously shown to perform relatively well compared to other pneumonia severity scores for predicting influenza‐related mortality [19], similar to our findings in this study. However, a retrospective study of patients admitted to a hospital in China with confirmed influenza reported discrimination against mortality of 0.78 for CURB‐65 and 0.56 for PSI [21], which differs from our observations. Less data are available regarding the predictive performance of the qSOFA, an index designed for rapid assessment of patients at risk for severe complications of sepsis, for severe respiratory disease. A retrospective 4‐year study of patients hospitalized with influenza in Switzerland determined a predictive accuracy of 0.89 for qSOFA against mortality, superior to both CURB‐65 (0.79) and PSI (0.79) [22]. Some studies have suggested a link between higher qSOFA scores and prolonged hospital stays and mortality in geriatric patients [11, 23]. Although we did not limit our analysis to older adults, the median age of our study population was 69 years, suggesting that our findings are likely representative of this older age group. In the context of these somewhat varied and conflicting results, the principal strengths of this study are twofold. First, the number of participants in this analysis is substantially greater than other studies examining these scores in hospitalized patients with confirmed influenza. Second, this study draws from a number of healthcare facilities across a wide catchment area, providing a much broader sample than studies with a more limited scope.

An important factor that may limit the application of validated disease severity scores in our analysis is the considerable heterogeneity that exists among adults hospitalized with influenza in the United States. Multiple patient and healthcare level factors, including baseline health status, socioeconomic status, and hospital admission practices, play a role in whether or not a patient will ultimately be hospitalized; these factors are largely unmeasured and unaccounted for in analyses of clinical surveillance data. Thus, a wide spectrum of illness severity likely exists among patients hospitalized with influenza in FluSurv‐NET that is not well characterized by age and underlying conditions. For example, an older person with multiple comorbidities might be hospitalized with relatively mild influenza, whereas younger individuals without medical comorbidities are less likely to be hospitalized without severe disease. Many existing scores, which take age and underlying conditions into account, may not adequately differentiate these two groups of individuals into low versus high risk for severe influenza outcomes. Additionally, in older adults, exacerbation of chronic conditions may contribute substantially to severe disease outcomes, limiting the performance of disease severity scores designed to predict outcomes from pneumonia or sepsis. An approach that first groups patients into classes based on objective measures of disease severity at admission, rather than relying on age and baseline health status, may allow for the development of scores that are better able to predict severe influenza‐associated outcomes [24].

There are several potential limitations to this study. First, influenza testing in FluSurv‐NET is clinician‐directed, and therefore subject to both institutional and provider‐associated testing biases. The 2017–2018 influenza season was predominated by Influenza A(H3N2) viruses in the United States and thus may not be representative of influenza seasons in which other viruses predominate. As our analysis was limited to a single season, it is possible that disease severity scores may perform differently in seasons with different circulating viruses or overall severity. Additionally, this study did not include outpatients or patients seen only in the emergency room, as all individuals captured by FluSurv‐NET have already been admitted to the hospital, and are therefore likely to be biased toward patients with more severe disease. Clinical pneumonia severity scores are often used to make decisions about whether a person requires hospital admission, therefore, individuals in our data set are likely to have higher scores than patients in the outpatient setting. Some individuals admitted with influenza may have required positive airway pressure for chronic conditions, such as sleep apnea, although we did not include positive pressure as a severe outcome if patients were already receiving this intervention at home prior to admission. The population enrolled in FluSurv‐NET may not be representative of the overall US hospitalized population, as the study catchment area only covers approximately 9% of the US population. Furthermore, the findings from this analysis may not be representative of all FluSurv‐NET patients as only a subset of patients were included in the disease severity project. Lastly, evidence of pleural effusion is a required element of the Pneumonia Severity Index; however, since this radiographic finding was not collected for FluSurv‐NET during the season included in our study, we revised PSI scores and risk tiers downwards for all participants.

In this large study of patients hospitalized with laboratory‐confirmed influenza, we found low to moderate discrimination overall for three widely used severity scores against a range of severe influenza‐related outcomes. As these scores are frequently used to make decisions regarding patient management and disposition, it is important to understand their limitations in the context of patients hospitalized with influenza. Given the immense annual burden of influenza disease in the United States and the wide range of clinical manifestations among patients hospitalized with influenza, this work underscores the need to better identify influenza‐specific indicators that predict severe outcomes.

## AUTHOR CONTRIBUTIONS

Conceptualization: Joshua Doyle, Shikha Garg, Carrie Reed, William Schaffner, H. Keipp Talbot, Wesley Self, Derek Williams, Evan Anderson, James Meek, Kimberly Yousey‐Hindes, and Ruth Lynfield. Formal analysis: Joshua Doyle, Alissa O'Halloran, and Lauren Grant. Investigation: Evan Anderson, Kyle Openo, Nisha Alden, Rachel Herlihy, James Meek, Kimberly Yousey‐Hindes, Maya Monroe, Sue Kim, Ruth Lynfield, Melissa McMahon, Alison Muse, Nancy Spina, Lourdes Irizarry, Salina Torres, Nancy Bennett, Maria Gaitan, and Mary Hill. Methodology: Joshua Doyle, Shikha Garg, Alissa O'Halloran, Lauren Grant, H. Keipp Talbot, Wesley Self, and Derek Williams. Project administration: Charisse Cummings. Supervision: Shikha Garg, Carrie Reed, William Schaffner, H. Keipp Talbot, Wesley Self, and Derek Williams. Validation: Alissa O'Halloran. Visualization: Joshua Doyle and Alissa O'Halloran. Writing: Joshua Doyle and Shikha Garg. Reviewing/Editing: Alissa O'Halloran, Lauren Grant, Shikha Garg, Carrie Reed, William Schaffner, H Talbot, Wesley Self, Derek Williams, Evan Anderson, James Meek, Kimberly Yousey‐Hindes, Ruth Lynfield, Kyle Openo, Nisha Alden, Rachel Herlihy, Maya Monroe, Sue Kim, Melissa McMahon, Alison Muse, Nancy Spina, Lourdes Irizarry, Salina Torres, Nancy Bennett, Maria Gaitan, and Mary Hill.

## CONFLICT OF INTEREST STATEMENT

E.J.A. has consulted for Pfizer, Sanofi Pasteur, GSK, Janssen, and Medscape, and his institution receives funds to conduct clinical research unrelated to this manuscript from MedImmune, Regeneron, PaxVax, Pfizer, GSK, Merck, Sanofi‐Pasteur, Janssen, and Micron. He also serves on a safety monitoring board for Kentucky BioProcessing, Inc., and Sanofi Pasteur. His institution has also received funding from NIH to conduct clinical trials of Moderna and Janssen COVID‐19 vaccines. E.J.A. is currently an employee of Moderna. All other authors declare no conflicts of interest.

### PEER REVIEW

The peer review history for this article is available at https://www.webofscience.com/api/gateway/wos/peer-review/10.1111/irv.13228.

## Supporting information


**Supplemental Table 1:** **Disease Severity Sampling Strategy by Site, FluSurv‐NET, 2017–18**
Supplemental Table 2: Components and Scoring Algorithms for CURB‐65 [5], Quick Sequential Organ Failure Assessment [4] and Pneumonia Severity Index [6]
**Supplemental Table 3. Comparison of Characteristics of Total Adult FluSurv‐NET Population versus Cases Included in the Disease Severity Analysis**.
**Supplemental Figure 1: Disease Severity Case Report Form, FluSurv‐NET, 2017–18.** This instrument was used in addition to the standard case report form to collect information regarding patient characteristics, vital signs, laboratory values, required clinical support modalities, and severe disease outcomes in the FluSurv‐NET network during the 2017–2018 influenza season.

## Data Availability

Individuals interested in receiving a limited dataset can submit a brief proposal to the corresponding author for review and consideration by the CDC and FluSurv‐NET site partners.
