# Supplementary material for: Performance of established disease severity scores in predicting severe outcomes among adults hospitalized with influenza—FluSurv‐NET, 2017–2018
Source: Influenza Other Respir Viruses. 2023 Dec 17;17(12):e13228. doi: 10.1111/irv.13228 (PMC10725795; doi:10.1111/irv.13228)

**Supplemental Table 1: Disease Severity Sampling Strategy by Site, FluSurv-NET, 2017-18**

| **Site** | **Sampling Scheme** | **Total number cases included in severity pilot** | **Total number of eligible FluSurv-NET cases^1^** | **Percent of cases included in severity pilot** |
| --- | --- | --- | --- | --- |
| CA | Did not participate | 0 | 1603 | 0% |
| CO | All cases admitted to 3 hospitals | 373 | 2017 | 18% |
| CT | All cases admitted to 2 hospitals | 711 | 1413 | 50% |
| GA | All cases admitted to 2 hospitals | 127 | 1358 | 9% |
| MD | All cases admitted to all hospitals | 2568 | 2568 | 100% |
| MI | All cases admitted to hospitals with remote EMR access | 1269 | 1455 | 87% |
| MN | Every fourth case admitted to hospitals with remote EMR access | 781 | 2181 | 36% |
| NM | Every fourth case admitted to all hospitals | 216 | 373 | 58% |
| NYA | All cases admitted to 2 hospitals | 136 | 522 | 26% |
| NYR | All cases admitted to hospitals with remote EMR access from Monroe County hospitals | 881 | 1895 | 46% |
| OH | Did not participate | 0 | 876 | 0% |
| OR | Did not participate | 0 | 603 | 0% |
| TN | All cases admitted to all hospitals | 1692 | 1692 | 100% |
| UT | All cases admitted to all hospitals | 870 | 870 | 100% |
| Total |  | 9624 | 19426 | 50% |

^1^ Includes total number of FluSurv-NET cases at each site who were sampled for complete medical chart abstraction. During the 2017-18 season, CA, GA, NM, OH, OR and NYA conducted medical chart abstractions on a 50% random sample of adults aged 50-64 years and a 25% random sample of adults aged 65 years and older. MN conducted medical chart abstractions on a 50% random sample of adults aged 65 years and older. For all other age groups and sites, medical chart abstractions were conducted on 100% of cases

**Supplemental Table 2: Components and Scoring Algorithms for CURB-65 [5], Quick Sequential Organ Failure Assessment [4] and Pneumonia Severity Index [6]**

| **Severity Score** | **Points Assigned** | **Score Range** |
| --- | --- | --- |
| **CURB-65** |  | 0-3 points |
| Confusion | +1 |  |
| Blood Urea Nitrogen (BUN) > 19 mg/dL | +1 |  |
| Respiratory Rate ≥ 30 | +1 |  |
| Systolic BP < 90 mmHg or Diastolic BP ≤ 60 mmgHG | +1 |  |
| Age ≥ 65 years | +1 |  |
| **Quick Sequential Organ Failure Assessment (QSOFA)** |  | 0 to 3 points |
| Altered Mental Status (Glasgow Coma Scale < 15) | +1 |  |
| Respiratory Rate ≥ 22 | +1 |  |
| Systolic Blood Pressure ≤ 100 | +1 |  |
| **Pneumonia Severity Index (PSI)** |  | Class I  Patient ≤ 50 years and  no coexisting illnesses  or physical examination findings |
| Age |  |  |
| Men | Age (yr) |  |
| Women | Age (yr) - 10 |  |
| Nursing Home Resident | +10 |  |
| Coexisting Illness |  | Class II  0-60 points |
| Neoplastic disease | +30 |  |
| Liver disease | +20 |  |
| Congestive heart failure | +10 |  |
| Cerebrovascular disease | +10 |  |
| Renal disease | +10 | Class III  61-80 points |
| Physical Examination Findings |  |  |
| Altered mental status | +20 |  |
| Respiratory rate ≥ 30/min | +20 |  |
| Systolic blood pressure < 90 mmHg | +20 |  |
| Temperature < 35°C or ≥ 40°C | +15 | Class IV  81-120 points |
| Pulse ≥ 125/min | +10 |  |
| Laboratory and Radiographic Findings |  |  |
| Arterial pH < 7.35 | +30 |  |
| BUN ≥ 30 mg/dL | +20 |  |
| Sodium < 130 mmol/L | +20 | Class V  121-305 points |
| Glucose ≥ 250 mg/dL | +10 |  |
| Hematocrit < 30% | +10 |  |
| Partial Pressure of Arterial Oxygen < 60 mmHg | +10 |  |
| **Pleural Effusion* | *+10* |  |
| *Pleural effusion data were not collected during the 2017-2018 season; all risk classes have been adjusted downward 10 points to compensate | | |

**Supplemental Table 3. Comparison of Characteristics of Total Adult FluSurv-NET Population versus Cases Included in the Disease Severity Analysis**.

|  | Total population of adults (n=27,523) | Sampled population of adults (n=19,428) | Data collected for severity project (n=9624) | Included in final analysis (n=8252) |
| --- | --- | --- | --- | --- |
|  | N (%) | N (%) | N (%) | N (%) |
| Median age (IQR) | 71 (58-82) | 71 (58-82) | 70 (57-82) | 71 (58-82) |
| Sex |  |  |  |  |
| Male | 12341 (44.8) | 8606 (44.8) | 4203 (44.3) | 3554 (43.0) |
| Female | 15182 (55.2) | 10822 (55.2) | 5421 (55.7) | 4698 (57.0) |
| Race/Ethnicity |  |  |  |  |
| NH White | 12513 (63.6) | 12312 (64.0) | 6280 (58.9) | 5354 (64.9) |
| NH Black | 3897 (19.8) | 3876 (18.7) | 2190 (27.9) | 1877 (21.6) |
| Hispanic | 1231 (6.3) | 1215 (6.3) | 547 (7.6) | 490 (7.1) |
| NH API | 719 (3.7) | 697 (5.1) | 182 (2.0) | 161 (2.0) |
| NH AIAN | 87 (0.4) | 36 (0.2) | 34 (0.5) | 31 (0.6) |
| Multiracial | 36 (0.2) | 36 (0.2) | 10 (0.1) | 7 (0.1) |
| Other/Unknown | 1204 (6.1) | 1197 (5.2) | 381 (3.0) | 332 (3.8) |
| Flu Type |  |  |  |  |
| Flu A | 19945 (72.5) | 14107 (71.8) | 7034 (73.0) | 5991 (73.2) |
| Flu B | 7442 (27.0) | 5225 (27.7) | 2541 (26.6) | 2221 (26.2) |
| Flu A/B | 107 (0.4) | 74 (0.4) | 34 (0.3) | 27 (0.4) |
| Unknown Type | 29 (0.1) | 22 (0.1) | 15 (0.1) | 13 (0.1) |
| In-hospital Death | 844 (3.1) | 844 (3.1) | 279 (4.2) | 183 (1.9) |
| Length of Stay, Median (IQR) | 3 (2-6) | 3 (2-6) | 3 (2-6) | 3 (2-5) |
| Site |  |  |  |  |
| CA | 3942 (14.3) | 1605 (14.3) | NA | NA |
| CO | 2017 (7.3) | 2017 (7.3) | 373 (10.0) | 349 (3.7) |
| CT | 1413 (5.1) | 1413 (5.1) | 711 (7.0) | 622 (6.6) |
| GA | 2766 (10.0) | 1358 (10.0) | 127 (13.7) | 108 (2.3) |
| MD | 2568 (9.3) | 2568 (9.3) | 2568 (12.7) | 2254 (23.9) |
| MI | 1455 (5.3) | 1455 (5.3) | 1269 (7.2) | 1130 (12.0) |
| MN | 3311 (12.0) | 2181 (12.0) | 781 (16.4) | 740 (13.1) |
| NM | 908 (3.3) | 373 (3.3) | 216 (4.5) | 199 (6.8) |
| NYA | 1248 (4.5) | 522 (4.5) | 136 (6.2) | 109 (2.5) |
| NYR | 1895 (6.9) | 1895 (6.9) | 881 (9.4) | 752 (8.0) |
| OH | 2007 (7.3) | 876 (7.3) | NA | NA |
| OR | 1431 (5.2) | 603 (5.2) | NA | NA |
| TN | 1692 (6.1) | 1692 (6.1) | 1692 (8.4) | 1211 (12.9) |
| UT | 870 (3.2) | 870 (3.2) | 870 (4.3) | 778 (8.3) |

**SUPPLEMENTAL FIGURE LEGENDS**

**Supplemental Figure 1: Disease Severity Case Report Form, FluSurv-NET, 2017-18.** This instrument was used in addition to the standard case report form to collect information regarding patient characteristics, vital signs, laboratory values, required clinical support modalities, and severe disease outcomes in the FluSurv-NET network during the 2017-2018 influenza season.

**Supplemental Figure 1: Disease Severity Case Report Form, FluSurv-NET, 2017-18**


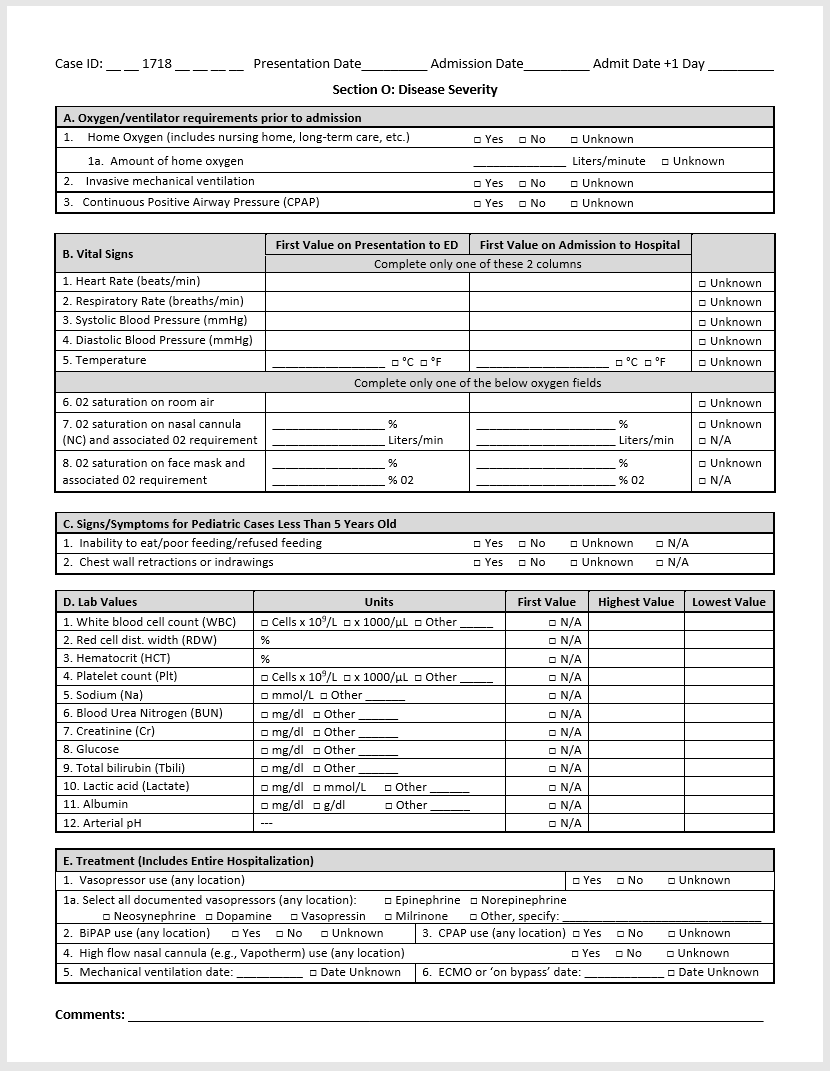

Supplement: Supplementary file 1 — Supplemental Table 1: Disease Severity Sampling Strategy by Site, FluSurv‐NET, 2017–18 Supplemental Table 2: Components and Scoring Algorithms for CURB‐65 [5], Quick Sequential Organ Failure Assessment [4] and Pneumonia Severity Index [6] Supplemental Table 3. Comparison of Characteristics of Total Adult FluSurv‐NET Population versus Cases Included in the Disease Severity Analysis. Supplemental Figure 1: Disease Severity Case Report Form, FluSurv‐NET, 2017–18. This instrument was used in addition to the standard case report form to collect information regarding patient characteristics, vital signs, laboratory values, required clinical support modalities, and severe disease outcomes in the FluSurv‐NET network during the 2017–2018 influenza season. [file IRV-17-e13228-s001.docx]
